# Piloting Implementation Design and Preliminary Readiness for Universal Suicide Risk Screening Program in Emergency Department of a Tertiary Care Centre, Nepal: A Mixed-Method Study

**DOI:** 10.31729/jnma.8832

**Published:** 2024-12-31

**Authors:** Anmol Purna Shrestha, Roshana Shrestha, Renu Shakya, Pratiksha Paudel, Madeleine Sorenson, Amrita Gurung, Riya Bajracharya, Ajay Risal, Lakshmi Vijayakumar, Ashley Hagaman

**Affiliations:** 1 Department of General Practice and Emergency Medicine, Kathmandu University School of Medical Sciences, Dhulikhel, Kavre, Nepal; 2 Department or Community Programs, Dhulikhel Hospital-Kathmandu University Hospital, Kavre, Nepal; 3 Sociaf and Behavioral Science Department, Yale School of Public Health, Connecticut, USA; 4 Department of Psychology, Padma Kanya Campus, Tribhuvan University, Nepal; 5 Department of Psychiatry, Dhulikhel Hospital-Kathmandu University Hospital, Kavre, Nepal; 6 Department of Psychiatry, Volunteer Health Services Hospital, Chennai, India

**Keywords:** *emergency medicine*, *developing world*, *implementation science*, *prevention*

## Abstract

**Introduction:**

Nearly three quarters of the suicides occur in developing world, however few evidence-based health systems strategies exist to detect and prevent suicide in these contexts. This pilot study evaluates the feasibility of implementing a universal suicide risk screening program in a Nepalese emergency department.

**Methods:**

This study reports the preliminary training phases of a pilot implementation trial in the emergency department to evaluate the program. The approval was obtained from the Nepal Health Research Council (Approval no. 447/2021 P), and the Kathmandu University School of Medical Sciences Institutional Ethical Review Board (Approval no. 237/2021) and Yale University IRB (Protocol ID 2000029480). Implementation assessments included suicide screening acceptability, appropriateness, confidence, system priority, and myth knowledge of staff. Implementation strategies were selected, decolonized, and preliminarily trained followed by phased supportive coaching to initiate the screening package. We designed the implementation package through co-design staff focus groups and embedded ethnography.

**Results:**

Co-design focus groups (n=8) occurred with staff and leadership. We trained 26 (76.47%) the staff on the Nepali suicide screening tool followed by supported phased initial screening over two months. Implementation assessments demonstrated increased scores on appropriateness, confidence, system priority, and myth knowledge. The implementation package included key strategies to be deployed over six months. The embedded ethnographic observations revealed barriers to effective implementation, such as anticipated stigma, reluctance to engage families, and distrust in referral processes.

**Conclusions:**

The pilot study demonstrated that training improves staff appropriateness, confidence, system priority, and myth knowledge. Despite initial barriers, co-designed strategies and phased coaching facilitates screening uptake, highlighting the program's potential for sustainable implementation.

## INTRODUCTION

Suicide is a global health issue with more than 700,000 deaths annually. Nepal has high suicide burden with an average of 14 people dying of suicide per day^1,2^ which increased during the COVID-19 outbreak.^3-5^ Dispelling complicated perceptions suicide causes, stigma, and legal implications is crucial to improve mental health services.^6^ Universal, selective, and indicative preventive strategies should be implemented to reduce the burden of suicide in Nepal.^7^ Emergency Departments (ED) is an important portal for identifying patients with suicide risk as individuals with comorbid conditions requiring emergency intervention and its associated costs may be at higher risk.^8^ Universal suicide screening in the medical setting^9,10^ and in the ED has proven to be feasible in developed world,^11-14^ However, EDs in Nepal are yet to implement suicide screening. The aim of this study is to implement design and readiness for a universal suicide screening program based on Ask Suicide-Screening Questions (ASQ)^15^ in the ED of our hospital.

## METHODS

The study was done in the high-volume ED of a tertiary care hospital in Nepal between November 2021 and August 2023. The hospital serves approximately 2.5 million people from Kavrepalanchok, Sindhupalchowk, Dolakha, Sindhuli, Ramechhap, Bhaktapur, and other surrounding districts. The ED is often at its 30-bed capacity, caring for 20,000 patient visits annually, many with high acuity. Most cases with suicidality present to the ED with about three suicide/self-harm cases per week, with seasonal peaks in the winter.^4^ The ED has 15-17 physician-level staff and 22 nursing/paramedic-level staff. The junior physicians, nurses, and paramedics were enrolled in the study with written informed consent.

**Figure 1:**
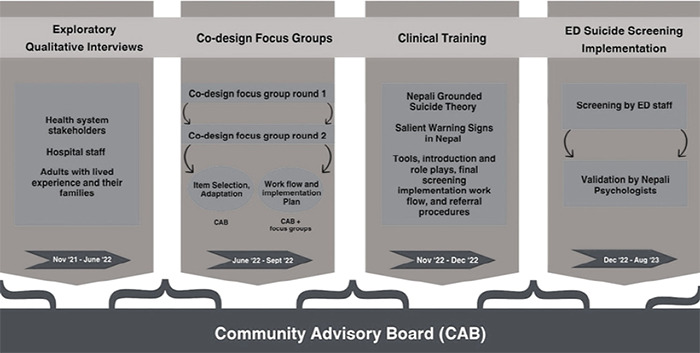
Suicide prevention package (SuPP) co-design process.

This study is part of the 'Integrating Suicide Prevention Packages into Task-Shifted Mental Health Interventions in Low-Resource Context' program co-designing a package of strategies to improve the integration of suicide prevention practices into the hospital system (Figure 1) (Clinical Trial ID: NCT06094959). We conducted a pilot implementation trial testing a package to support universal screening and referral using the culturally adapted Ask Suicide Questionnaire. We used mixed methods and the EPIS (exploration, preparation, implementation, and sustainment) framework to design, adapt, and assess the feasibility and acceptability of universal screening for suicide risk among staff and patients.

Institutional Review Board approval was obtained from Yale University IRB Protocol ID 2000029480), the Nepal Health Research Council (Approval no. 447/2021 P), and the KUSMS institutional ethical review board (Approval no. 237/2021). The co-design process began with an exploratory phase to assess staff and institutional readiness for suicide screening. The qualitative methods in this phase included three main components: ethnographic observation, individual in-depth interviews, and focus group discussions (FGDs) over 6 months (April-November 2022).

This phase also involved mapping out the current implementation pathways to assess the efficacy of ongoing strategies and envision improved systems for future enhancement of suicide prevention efforts. The findings of the mixed-methods convergent design and triangulated results to form conclusions and recommendations for the next phase is under review for publication in a separate paper.

Among several validated screening tools available,^16^ we used a tool based on the ASQ questionnaire.^15^ We took a systematic approach to transcultural translation and adaptation to optimize equivalence to the original ASQ tool^15^ including comprehensibility, relevance, completeness, and acceptability of the response set.^6,17^ The ASQ was originally a 4-item questionnaire. Due to the double-barreled nature of item 2,^18^ it has been separated into two distinct items. Thus the validated Nepali ASQ consists of 5 items derived from the original ASQ questionnaire.^19^ The Nepali ASQ screening tool was built into an application compatible with mobile devices. Every ED staff willing to screen patients was given a unique code to log in to the application. After completion of screening of an individual patient, the data was automatically synced and filled in the database once the device had internet access.

The Nepali ASQ tool consists of five yes/no questions and takes about 3 minutes to administer. The risk was stratified as positive response to any of the initial 5 items and categorized as negative screening and coded green for negative response to all 5 items. The patients with negative responses were discharged with the resource card (supplement 2). If positive response, items 6-8 was asked and if "yes" to any of the items, screening was categorized as high risk and coded red. If negative response to items 6-8, the screening was categorized as low risk and coded yellow. This is equivalent to Brief Suicide Safety Assessment (BSSA) as recommended in the ASQ workflow.^20^ The patient identified as having low risk for suicide was given the resource card and referred to outpatient mental health service during discharge. For the patients having high risk for suicide, an on-duty psychiatrist was called for further mental health/suicide safety assessment at the ED or the psychiatric consultation room.

We adapted the Zero Suicide Workforce Survey to assess screening implementation within the zero suicide initiative.^21^ We assessed 5 domains: acceptability (5-items), appropriateness(6-items), individual confidence (8-items), hospital priorities (6-items), and myths knowledge about suicide prevention (4-items) derived from our formative qualitative interviews and the Community Advisory Board (CAB). We conducted baseline and post-training AACPK assessments.

The implementation package was designed through co-design focus groups with ED staff and embedded ethnography. The assigned research team members observed the participants, current clinical workflows and potential barriers and facilitators, shadowed the ED staff and analyzed the completed screening documents. Implementation strategies were selected, decolonized, and preliminarily trained through initial training followed by phased supportive coaching to initiate the screening package. Between November 2022 and December 2022, we trained ED staff for screening in two batches on a voluntary basis. This training included introducing the Nepali ASQ tool, role-playing exercises, and finalizing the screening workflow. We present the results of the training and co-designed implementation package.

For socio-demographic characteristics, we presented frequency and percentage. For the AACPK assessment, the scoring for negative items was done in the reverse order. For the acceptability domain, there were a total of six items and the score ranged from 0 to 2. Two items in acceptability domain were reverse-coded. In the appropriateness domain, there were a total of six items. The score ranged from 0 to 3. '0' Never, '1' Rarely, '2' Sometimes, and '3' Always. For the confidence domain, there were a total of 7 items. The score ranged from 0 to 3. One item in the confidence domain was reverse-coded. There was a total of 6 items in the system priority domain and the score ranged from 0 to 3. Finally, for the myth knowledge domain, there were a total of 4 items ranging from score 0 to 1. All the items in this domain were reverse-coded. We calculated the mean and standard deviation for each domain and item, respectively.

The study team conducted focus groups and embedded ethnographic observations over a six-month period. The interviewers and ethnographers took field notes in English and held debriefing sessions following each FGD. All audio recorded data were first transcribed into Nepali with an English translation immediately following. Culturally salient idioms and concepts remained intact in the original language. Quality and trustworthiness were established through multiple quality checks of the transcripts with multiple study team members, daily debriefs and member checking with clinical ED staff. Transcripts were analyzed thematically, first inductively to attend to grounded concepts, framings, and articulated barriers and then abductively integrated with broad domains of Consolidated Framework for Implementation Research (CFIR) 2.0 that were salient to the study's context. The team then combined insights from the field notes, codes, and memos to identify analytic themes. They further refined these themes through collaborative discussions with the authorship team, including cowriting sessions and brief meetings with emergency department staff, leading to the final presentation of the main themes. Qualitative analysis was conducted using NVivo.

## RESULTS

Out of 34 staffs, 26 (76.47%) ED staff voluntarily enrolled in the study for training on the use of the Nepali ASQ tool. The staff were trained in two batches as all staff couldn't be released from duty in one sitting. Eighteen (69.23%) were of the age group 25-34 years, with the majority of the enrolled staff being female 18 (69.23%) (Table 1).

**Table 1:** Demography of training participants enrolled on the use of Nepali ASQ tool (n=26).

Characteristics	Baseline characters n(%)
**Age (years)**	
18-24	5 (19.23)
25-34	18 (69.23)
35-44	2 (7.69)
45-54	1 (3.85)
**Sex**	
Female	18 (69.23)
Male	8 (30.77)
**Job Title**	
Nurse/Paramedic	16 (61.54)
Medical Officer (MBBS)	8 (30.77)
Faculty (MD)	1 (3.85)
Intern (One month clerkship in ED)	1 (3.85)
**Years Spent in Healthcare**	
0 – 5	18 (69.23)
6 – 10	4 (15.38)
11 – 15	2 (7.69)
16 +	2 (7.69)

While comparing pre- and post-training, on average, the score for appropriateness of suicide screening increased by 4 units, the score for individual confidence increased by 4.30 units, the score for system priorities increased by 2.03 units the score for suicide myth knowledge increased by 0.73 units after training. However, there was no change in the acceptability of suicide screening tool (Table 2,3).

**Table 2:** The pre- and post-training comparison of the AACPK score of all the items (n=26).

Q. No	Question	Scoring	Avg. pre-Score	Avg. post-Score
**A**	**Acceptability**		
1	Suicide screening tools can identify more than half of patients with suicidal thoughts and symptoms behaviors than more informal methods	0 - 2	1.46	1.88
2	Suicide screening tools can ensure that all clinicians ask every patient the same questions, giving each at-risk client the same opportunity to be identified	0 - 2	1.53	1.81
3	Suicide screening tools can negatively affect rapport and trust with clients	0 - 2	1.31	1.38
4	Suicide screening tools are not necessary for senior clinicians who have experience and expertise with mental health assessment	0 - 2	1.58	1.54
5	Suicide screening tools allow different clinicians to communicate more clearly and have a uniform vocabulary for documentation	0 - 2	1.81	1.92
**B**	**Appropriateness**		
1	I understand my role and responsibilities related to suicide prevention within Dhulikhel Hospital's Emergency Department	0 - 3	2.42	3.00
2	I believe suicide prevention is a part of my regular clinical work	0 - 3	2.27	2.81
3	I currently screen patients for suicide risk (screening refers to asking a set of questions about suicide to patients)	0 - 3	1.38	1.77
4	I know what procedures to follow when I suspect that an individual may be at risk for suicide	0 - 3	1.65	2.77
5	I have the time to dedicate to screening patients for suicide risk given all of my other responsibilities	0 - 3	1.81	2.27
6	I receive enough supportive supervision to effectively assist patients with suicidal thoughts and behaviors, especially if I am unsure of what to do	0 - 3	1.65	2.58
**C**	**Individual confidence**		
1	I am confident that I can identify if a patient is at risk for suicide	0 - 3	1.88	2.54
2	I am hesitant to ask patients directly about their suicidal thoughts and behaviors	0-3	1.54	1.65
3	I feel confident in my ability to get patients to talk about their suicidality honestly	0-3	1.81	2.50
4	I feel confident in my ability to talk to families about how they can help their family member if they are at risk for suicide	0-3	2.00	2.50
5	I feel confident in my ability to help suicidal patients connect with mental health services that are accessible and affordable for them	0-3	2.11	2.57
6	I am comfortable asking my colleagues for help in managing suicidal patients without feeling embarrassed or burdensome	0 - 3	2.42	2.62
7	I feel very confident to counsel patients or their families to remove or lock up any pesticide or medication at home when patient has thoughts of suicide	0-3	2.04	2.65
8	I have the knowledge and skills needed to screen individuals for suicide risk	0-3	1.65	2.73
**D**	**Hospital priorities**			
1	This hospital has policies and procedures in place that clearly define each employee's role in preventing suicide	0-3	1.79	1.85
2	Leadership in my department makes suicide prevention a priority	0-3	1.92	2.46
3	The psychiatry department is easy to collaborate with when there is a suicide case	0-3	1.73	1.73
4	If I need to spend more time to support suicidal patients, my supervisor is supportive to manage my other responsibilities	0 - 3	2.08	2.42
5	Is there a belief in the hospital that universal screening is possible in their everyday work?	0-3	1.62	2.19
6	Is there a belief in the hospital that suicide prevention is an important part of everyone's job?	0-3	2.13	2.65
**E**	**Myth knowledge**			
1	If I talk to someone about suicide, I might unintentionally "put the idea in their head" or trigger them to consider it	0 - 1	0.54	0.81
2	People who really want to kill themselves wouldn't tell someone they are thinking about suicide	0 - 1	0.42	0.46
3	If someone wants to kill themselves, there is not much anyone can do about it	0 - 1	0.61	0.73
4	Most suicides occur with little or no warning signs	0 - 1	0.27	0.58

**Table 3:** The pre- and post-training comparison of the AACPK score of the domains.

Characteristics	Post-training		Pre-training		Differences	
Overall (n=26)	Mean	SE	Mean	SE	Mean	SE
Acceptability	8.5	0.32	7.69	0.45	0.84	0.48
Appropriateness	15.19	0.46	11.19	0.63	4	0.72
Confidence	19.76	0.57	15.46	0.57	4.30	0.86
System priority	13.31	0.73	11.27	0.92	2.03	0.88
Myth	2.58	0.24	1.84	0.24	0.73	0.25

**Implementation blueprint and strategy bundle development:**

**Figure 2:**
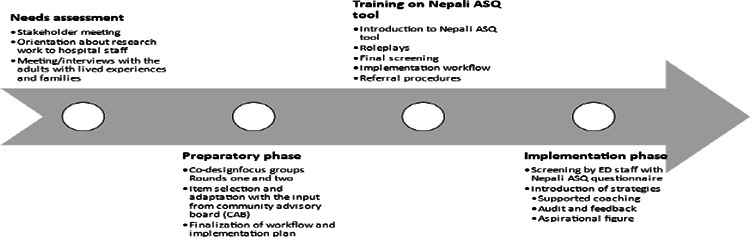
Implementation blueprint.

We used embedded ethnography to observe current clinical workflows and potential barriers and facilitators. We also used co-design focus groups and dynamic training to develop the Nepali ASQ workflow and implementation blueprint (Figure 2). We also used co-design focus groups to develop a preliminary implementation strategy bundle of the five strategies (Table 4).

**Qualitative findings informing implementation strategy development:**

FGDs with frontline health workers revealed important perceived barriers to universal screening implementation that informed the co-design of our implementation strategies. While health staff reported deep concern for addressing the issue of suicide, they expressed concern when planning the blueprint of the screening protocol given various contextual, system, team dynamic, and individual level barriers. Team communication barriers where perceived hierarchies and communication practices result in overburdened health staff and limited and challenging intra-departmental collaboration. System feasibility barriers where a lack of workflows, protocols, and standardized training in place for managing suicide cases results in disjointed workflows and low confidence in screening and support capability of staff to identify and support patients. Social stigma barriers where staff perceptions of suicidal patients result in stigmatized conversations about suicide among health staff. Finally, task priority barriers where ED staff emphasize their role to stabilize and refer patients as their main priorities and expressed concern for the ability to prioritize the time needed for screening and engaging in a complex topic like suicide.

We infer the following targets from the health staff interviews to design implementation strategies in order optimizing the suicide prevention package. We provide qualitative quotes to contextualize these strategy targets:

1. Staff lack of confidence and experience handling suicidal patients and their families *“I can talk or ask about it and…maybe I can find what is happening. But like I said, I feel like I can’t do anything to prevent it…it is called that self what [slightly laughing] I have a lot of self-doubt.”*

2. Staff communication during handover is challenging and monitoring is essential

3. Suicide prevention is important, but who will do it? There is a need for a clearly written clinical protocol and workflow that is feasible. *“I am interested. If you ask me, I would have done it. But I don't have time for it, right? In my opinion, if it does good to the patient, a doctor would never say no to it. WE should have dedicated staff. Without dedicated staff, it would be difficult to do even though the idea is very good.”*

4. Staff have both extensive clinical exposure and personal exposure to suicide *"Some of the patients that come, they are calling for attention by having some stress…Sometimes self-harm behavior comes into our ER, but they don’t have any suicidal intent. When the suicide patient comes, and when I go there, they are in an ambiguous state, we don’t know how likely they are to do anything….and the families, like I already told you, if a family member is with a patient and I’m asking the patient if this was a suicide attempt, the family say, “No that has not happened! Who told you this! Nothing like that has happened (in high pitch)!” The family do not let the patient speak and if I say that the patient might have been thinking of suicide, the family thinks that now I gave them this idea.”*

5. Suicide screening is acceptable and feasible. But the implementation strategy bundle needs to address stigma and appropriateness. *“I’ve heard other staff calling those suicidal patients ‘psycho’. Sometimes staff joke about those patients, they’ll say if they really wanted to die, why did they only take a little bit of poison? I hear those things."*

**Table 4:** Implementation strategies.

Domain	Strategy 1: Supportive supervision	Strategy 2: Credential standard	Strategy 3: Audit and feedback	Strategy 4: Ongoing training and coaching	Strategy 5: Developed a formal implementation blueprint
Actor	Clinical psychologist or psychological nurse specialist	Hospital system alongside the academic arm of the hospital	Implementation lead	Implementation lead and clinical psychologist on staff	ED clinical staff
Action	Supportive supervision meetings	Codify standards to achieve clinical certification as a 'Suicide Prevention Specialist'	-Online weekly messages -Biweekly feedback meets	-Supportive coaching for screening at ED -Aspirational figures -Dynamic training to onboard new clinicians	Created a workflow
Target of action	ED staff newly trained in screening with Nepali ASQ tool	Motivation to screen, attend meetings and to sustain screening over time.	-Identify and prepare champions -Identify early adopters -Develop partnership -Optimism and aspiration that the Nepali ASQ screening will be effective	Initial training in screening implementation and workflow procedures, identify barriers and facilitators, support role plays and model high fidelity, support selfefficacy, inspire and motivate.	Document clear roles and responsibilities and how to implement each component
Temporality	Dec 2022- Aug 2023	Certification given after completing all standards	Nov 2022- Aug 2023	Dec 2022- Aug 2023	Nov 2022 with adaptations as needed
Dose	Biweekly over lunch break	N/A	Weekly from Nov 2022 to March 2023, then ad hoc afterwards	Biweekly	Initial mapping, design, and training with updates as needed
Implementation outcome affected	Acceptability, Appropriateness, Confidenc	Screening Uptake, system priorities	Uptake, Acceptability	Acceptability, Appropriateness, Confidence	Acceptability, Appropriateness, Confidence, system priorities
Justification	Formative data suggested initial clinician hesitancy, stigma, concern for role-fit, and concern for patient response	- Credentialing allows for hospital priority demonstration, rigor and fidelity, and institutional support for the practice. - Credentialing may improve motivation and sustainment of practice	Receiving information that current clinical practice is inconsistent with desired practice, guidelines, and peer implementation allows focus to shift to troubleshooting improvements and behavior change	Social learning theory, research suggests that ongoing training and coaching is more effective compared to single event trainings	Formative data suggested initial clinician confusion on role clarity, concern for time, concern for referral efficiency

## DISCUSSION

Following the FGDs and embedded ethnography, we designed the implementation strategy package to roll out with the universal suicide screening program (Table 4). The first strategy was supportive supervision. Supervision meetings were held over lunch breaks for ED staff regardless of screening uptake. The second strategy was creation of credentialing standards. The criteria for eligibility for certification were: completion in person 5-hour long training, screening continuously over six months in a patient care setting, engagement in weekly/monthly supportive supervision sessions and completion of 100 suicide screens at the implementation phase. The criteria for certification were approved by the training lead of the training center at the hospital. The third strategy involved provision of audit and feedback via online messages. Online messages about weekly screening progress which helped identify early adopters (staff who screened the most weekly) which in turn motivated other staff to screen. In addition to this, in-person biweekly feedback meets over lunch were the activities done as per the strategy. Supportive coaching and ongoing training involved the fourth strategy. Finally, a formal implementation blueprint was formulated. outlining what staff were responsible for various parts of screening process (identifying patients, documenting results, initiating referral procedures)

Our study investigated the implementation design and preliminary readiness of ED staff for universal suicide screening at our ED. We trained 26 (76.47%) ED staff on the Nepali ASQ tool followed by supported phased initial screening. Implementation assessments demonstrated increased scores on appropriateness, confidence, system priority, and myth knowledge. Research indicates that ED staff generally support suicide prevention initiatives, although attitudes and levels of confidence can differ based on specific roles and settings. ED nurse leaders show strong support for lethal means counseling, but many remain skeptical about the preventability of suicide.^22^ The findings from the study by Betz et al also highlight the need to address and modify misperceptions about the preventability of suicide in efforts to implement and sustain lethal-means counseling practices in emergency departments. Likewise, behavioral health providers and ED staff reported higher confidence in suicide-related care compared to other roles in another study.^23^

We described the implementation strategies deployed over six months. The embedded ethnographic observations revealed barriers to effective implementation. Perceived hierarchies and communication practices result in overburdened health staff and limited and challenging intra-departmental collaboration. Disjointed workflow and low confidence in screening among staff was due to lack of protocols, and standardized training. Studies show that barriers and challenges to implementation can be addressed using various interventions. The study by Chesin et. al. show that Safety Planning Intervention with Structured Follow-Up (SPI-SFU) is perceived as helpful in connecting patients to services.^25^ The implementation of the SPI-SFU intervention increased staff comfort in discharging veterans at some suicide risk. Similarly, the SAFE VET intervention, combining safety planning with follow-up, was found acceptable and useful by participants.^25^ The overwhelming majority of participants found the intervention to be very acceptable and would recommend it to others in crisis. Attitudes toward continuous quality improvement in suicide care are more positive among younger, less experienced staff, administrators, and those in ED or outpatient settings.^23^ These findings highlight the importance of role-specific training and implementation strategies for suicide prevention in EDs.

The FGDs with the staff highlighted the importance of suicide screening, although they pointed out the lack of designated staff for the same. South Asian countries face challenges regarding suicide prevention due to limited knowledge and training among ED staff. Study reveal that many ED staff in Pakistan lack awareness of suicide-related laws and specific training in managing suicidal patients.^26^ Decisionmaking for patients in suicidal crisis is affected by patient factors, contextual factors, and staff-related factors, highlighting the need for additional training in suicide prevention.^27^ Common suicide methods in this part of the world include hanging and poisoning, with hanging becoming more prevalent in recent years.^28^ To address these issues, suicide prevention programs (SPPs) are being developed to improve knowledge, attitudes, and gatekeeper behavior among healthcare professionals.^29^ Implementing and evaluating such programs could potentially reduce suicide rates among college students and other vulnerable populations in South Asian countries.

Research in high-income settings indicates that ED staff often hold misconceptions about suicide prevention and may lack confidence in supporting suicidal patients. A study found that many ED providers believed most Golden Gate Bridge jumpers would have found alternative means if barriers were present, highlighting a common myth.^30^ Similarly, despite recognizing the importance of asking suicidal patients about firearm access, many ED staff rarely do so.^30^

However, targeted training can improve attitudes and perceived competence in suicide prevention among ED nurses.^31^-^32^ A workshop in Japan demonstrated positive changes in nurses' understanding and willingness to care for suicidal patients.^31^ Similarly, a study in the UK found a correlation between suicide prevention training and nurses' perceived competence in triaging suicidal individuals.^32^

Enhanced education and training are needed to address misconceptions and improve suicide prevention practices in emergency settings. Researched also indicate the need for strategies beyond training events to increase uptake of prevention integration in emergency settings. In complex resource limited environments, such strategies may be more significant given limited human resources and limited technology.

As this study reports the results of the preparatory and early implementation phase of a larger package, the findings need to be further correlated with those done in the 3 and 6 months. Additionally, since the enrollment of the ED staff was on voluntary basis, we couldn't make the screening universal.

## CONCLUSIONS

Suicide is a complex event with little histories of health system prevention in Nepal. Our preliminary implementation co-design work suggests that suicide screening is feasible and acceptable, but will require a robust implementation strategy bundle to support its deployment given resource-strained infrastructure, challenging working environments, no previous legacies of mental health care integration into emergency departments, and larger social and clinical stigma surrounding engaging the topic of suicide. This study outlines salient implementation strategies that may be supportive of implementing much needed screening and referral processes to begin to address the growing burden of suicide in Nepal.
